# Targeting ferroptosis: a promising strategy to overcome drug resistance in breast cancer

**DOI:** 10.3389/fonc.2024.1499125

**Published:** 2024-12-20

**Authors:** Cuixin Peng, Yanning Chen, Mingzhang Jiang

**Affiliations:** Department of Pharmacy, West China Xiamen Hospital of Sichuan University, Xiamen, China

**Keywords:** ferroptosis, breast cancer, chemotherapy resistance, iron metabolism, antitumor mechanism

## Abstract

Breast cancer is one of the most prevalent malignancies affecting women worldwide, with its incidence increasingly observed in younger populations. In recent years, drug resistance has emerged as a significant challenge in the treatment of breast cancer, making it a central focus of contemporary research aimed at identifying strategies to overcome this issue. Growing evidence indicates that inducing ferroptosis through various mechanisms, particularly by inhibiting System Xc^-^, depleting glutathione (GSH), and inactivating glutathione peroxidase 4 (GPX4), holds great potential in overcoming drug resistance in breast cancer. It is anticipated that therapies targeting ferroptosis will emerge as a promising strategy to reverse tumor resistance, offering new hope for breast cancer patients. This review will explore the latest advancements in understanding ferroptosis in the context of breast cancer drug resistance, with a particular emphasis on the roles of ferroptosis inducers and inhibitors, and the impact of ferroptotic pathways on overcoming drug resistance in breast cancer.

## 1 Introduction

Ferroptosis, a form of regulated cell death characterized by the accumulation of iron-dependent lipid reactive oxygen species (Lip-ROS), has garnered significant attention in the scientific community since its initial description by Dixon et al. in 2012 (1). Distinct from traditional forms of cell death such as apoptosis, necroptosis, autophagy, and pyroptosis, the fundamental mechanism of ferroptosis involves iron overload, which leads to dysregulated lipid peroxidation and excessive accumulation of reactive oxygen species (ROS). This process ultimately triggers cell death, characterized by specific morphological features including mitochondrial crumpling, reduction or disappearance of mitochondrial cristae, increased mitochondrial membrane density, and rupture of the mitochondrial membrane (2).

**Figure 1:**
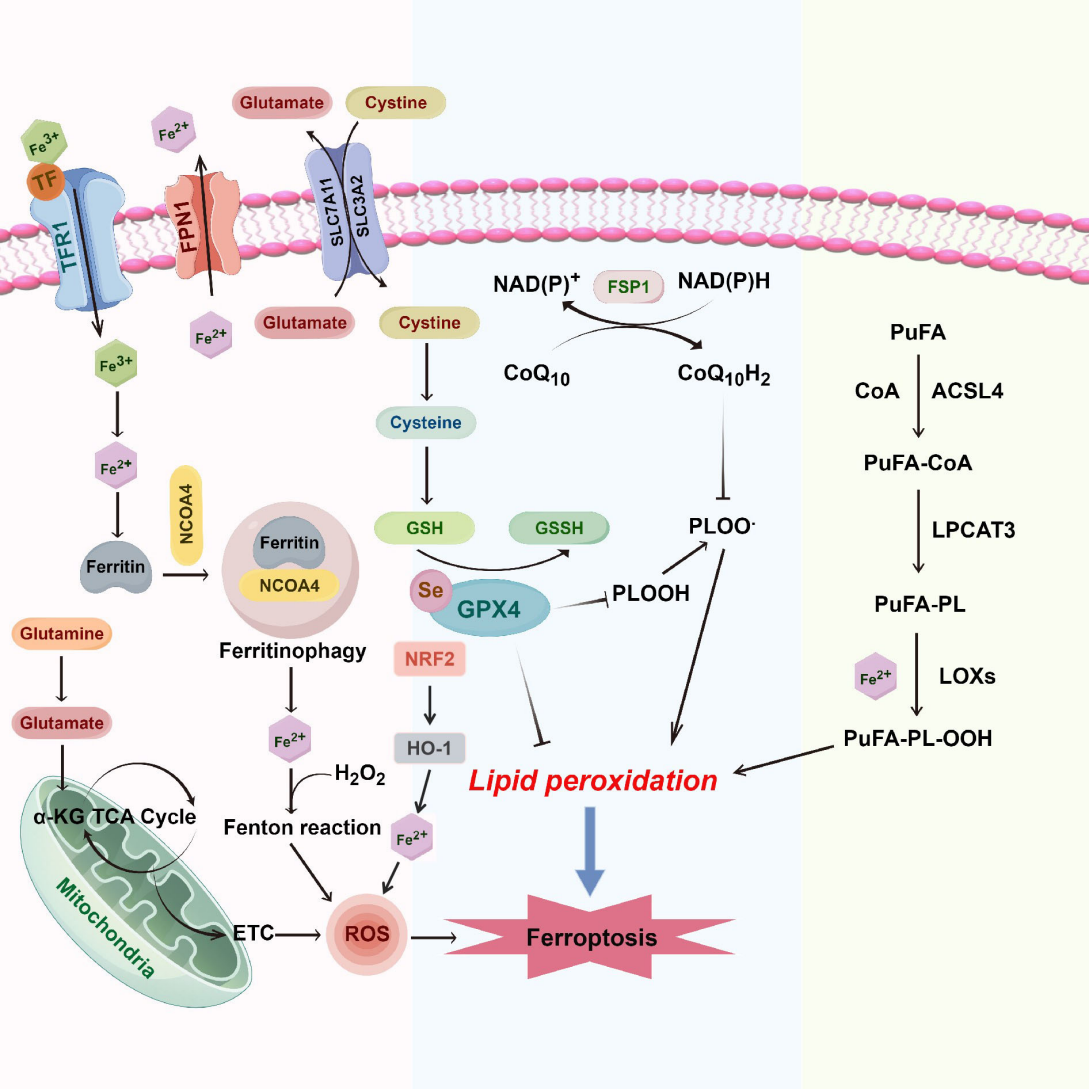
Molecular mechanisms of ferroptosis in breast cancer. Ferroptosis in breast cancer is driven by the intricate interplay between iron metabolism, oxidative stress, and lipid peroxidation. System Xc^-^ (SLC7A11/SLC3A2) facilitates the uptake of cystine in exchange for glutamate, supporting GSH synthesis. GPX4 employs GSH to reduce lipid hydroperoxides (PUFA-OOH) to lipid alcohols (PUFA-OH), mitigating lipid peroxidation. Excess intracellular ferrous iron (Fe^2+^) via the transferrin receptor (TFR1) and heme oxygenase-1 (HO-1) generates ROS, which foster further lipid peroxidation through the Fenton reaction and mitochondrial electron transport chain (ETC). FPN1 is the only known iron exporter in mammals and plays a vital role in maintaining systemic iron balance. In the context of ferroptosis, FPN1’s regulatory function impacts intracellular iron levels, which are critical in the generation of ROS via the Fenton reaction. By exporting excess iron, FPN1 helps mitigate the risk of iron-induced oxidative stress and lipid peroxidation, thus influencing ferroptosis susceptibility in breast cancer cells. Accumulating PUFA-PLs, synthesized by ACSL4 and LPCAT3, are peroxidized by lipoxygenases (LOXs), forming PUFA-PL-OOH, culminating in ferroptosis. The FSP1/CoQ10 axis further regulates ferroptosis by utilizing NAD(P)H to regenerate reduced CoQ10, which acts as an antioxidant to inhibit lipid peroxidation and protect against iron-induced oxidative stress. α-KG, alpha-ketoglutarate; ACSL4, acyl-coA synthetase long-chain family member 4; CoA, coenzyme a; ETC, electron transport chain; FPN1, ferroportin 1; GPX4, glutathione peroxidase 4; GSH, glutathione; GSSH, oxidized glutathione; HO-1, heme oxygenase 1; LPCAT3, lysophosphatidylcholine acyltransferase 3; LOXs, lipoxygenases; NRF2, nuclear factor (erythroid-derived 2)-like 2; PUFA, polyunsaturated fatty acids; PUFA-OOH, lipid hydroperoxides; PUFA-PL, phospholipid-bound polyunsaturated fatty acids; ROS, reactive oxygen species; SLC7A11, solute carrier family 7 member 11; SLC3A2, solute carrier family 3 member 2; TCA cycle, tricarboxylic acid cycle (krebs cycle); TFR1, transferrin receptor 1; TF, transferrin; FSP1, ferroptosis suppressor protein 1; CoQ10, coenzyme Q10.

Recent studies have underscored the pivotal role of ferroptosis in the pathogenesis of various human diseases and its strong association with therapeutic efficacy in cancer treatment, particularly in breast cancer (**Figure 1**), the most common malignancy among women worldwide (3, 4). As one of the leading causes of cancer-related mortality in women, breast cancer has exhibited an alarming trend of earlier onset, posing an escalating threat to women’s health (5). Overcoming or mitigating resistance to chemotherapy and molecular targeted therapies remains a major challenge in the field, critically impacting the overall effectiveness of breast cancer treatment (4).

In this context, ferroptosis has emerged as a potential strategy to combat breast cancer drug resistance. A burgeoning body of evidence suggests that ferroptosis can be induced in tumor cells through inhibition of System Xc^-^, depletion of glutathione (GSH), and inactivation of glutathione peroxidase 4 (GPX4), thereby counteracting the mechanisms of drug resistance in breast cancer (6). This review will examine the role of ferroptosis in breast cancer drug resistance, summarize the current state of research, and explore its potential for therapeutic application in overcoming treatment resistance.

## 2 Inducers and inhibitors of ferroptosis

Ferroptosis inducers and inhibitors play a pivotal role in influencing tumor therapy and overcoming chemotherapy resistance. Type I ferroptosis inducers, such as erastin, sulfasalazine, and sorafenib, initiate ferroptosis by targeting System Xc^-^ and obstructing the amino acid transporter necessary for cysteine import, which is crucial for glutathione synthesis. Additionally, type II ferroptosis inducers, including RSL3 and ML210, have been shown to induce ferroptosis primarily by inhibiting GPX4. A recent study further revealed that TIMM17B may impact ferroptosis and chemotherapy resistance in breast cancer through its effect on GPX4 (7). Other inhibitors include buthionine-sulfoximine (targeting GSH), α-tocopherol (targeting Lip-ROS), DFO (targeting the Fenton reaction), ferrostatin-1 (targeting Lip-ROS), and liproxstatin-1 (targeting Lip-ROS) (6).

Studies have demonstrated that erastin significantly enhances the chemotherapeutic efficacy of temozolomide, cisplatin, cytarabine, and doxorubicin by inducing ferroptosis (8). Neratinib, an irreversible pan-tyrosine kinase inhibitor and ferroptosis inducer, has been shown to effectively inhibit breast cancer growth and metastasis in highly metastatic breast cancer cell models (9). In addition, various compounds or extracts, including curcumin, metformin, and lidocaine, have been reported to enhance therapeutic efficacy against breast cancer through the ferroptosis pathway (10–12). Furthermore, Wang et al. found that isoliquiritin could regulate ferroptosis and ameliorate resistance to doxorubicin via the NF-κB signaling pathway in breast cancer (13). Overall, the development of effective drugs that induce ferroptosis in breast cancer cells is expected to improve treatment outcomes and offer new hope for breast cancer patients, particularly those resistant to chemotherapy (14).

## 3 Ferroptosis-related drug resistance in different subtypes of breast cancer

Chemotherapy is a critical therapeutic strategy for breast cancer, which remains a complex process involving multiple genes and signaling pathways. At present, chemotherapy resistance represents a major obstacle to the efficacy of treatment, making it an urgent issue to address. Extensive evidence has shown that chemoresistance is linked to various signaling pathways, including apoptosis inhibition, enhanced drug efflux, increased oxidative stress defenses, epithelial-mesenchymal transition, and the regulation of non-coding RNAs in tumor cells (14). Ferroptosis-associated drug resistance varies across different molecular subtypes of breast cancer, with triple-negative breast cancer (TNBC) being particularly prone to recurrence due to cellular resistance (15). The high mortality rate of metastatic TNBC is largely attributed to the presence of drug-resistant breast cancer stem-like cells (BCSCs), in which ferroptosis is significantly suppressed. Modulating iron metabolism has emerged as a potential strategy to regulate chemotherapy resistance in breast cancer (16).

Yang et al. demonstrated that phenazine derivatives can induce ferroptosis in BCSCs by modulating iron metabolism, resulting in anticancer effects (17). Similarly, Mai et al. successfully induced BCSC death by manipulating iron metabolism using ironomycin (16). Wu et al. developed iron nanoparticles loaded with siProminin2, which promoted ferroptosis in BCSCs by regulating iron metabolism and GPX4, thereby achieving antitumor effects (18). Additionally, Liang et al. identified heat shock protein beta-1 (HSPB1) as a critical drug resistance factor in TNBC, with its expression correlating with poor prognosis. Up-regulation of HSPB1 may overcome doxorubicin resistance by regulating ferroptosis (19). Recent studies also revealed that hepatic leukemia factor (HLF) can promote chemoresistance in TNBC by inhibiting ferroptosis through the regulation of the IL-6/TGF-β1 signaling axis, ultimately facilitating tumor progression and metastasis (20). The suppressor of cytokine signaling 1 (SOCS1) has been shown to regulate ferroptosis in TNBC by modulating GPX4 expression, inhibiting cell proliferation, and contributing to cisplatin resistance (21).

Collectively, these studies underscore the pivotal role of ferroptosis in regulating tumor survival and drug resistance in TNBC via multiple mechanisms. In HER2-positive breast cancer, tyrosine kinase inhibitors (TKIs) have been shown to significantly affect chemotherapy resistance through ferroptosis. Nagpal et al. recently discovered that integrin αvβ3 mediates chemotherapy resistance in HER2-positive breast cancer via TKIs in both mouse and human models. This resistance can be reversed through gene amplification or pharmacological approaches aimed at restoring ferroptosis sensitivity, potentially offering new therapeutic strategies for chemotherapy-resistant HER2-positive breast cancer (22). Similarly, Zou et al. demonstrated that inhibition of fibroblast growth factor receptor 4 (FGFR4) can trigger ferroptosis in breast cancer cells through the β-catenin/TCF4-SLC7A11/FPN1 axis, presenting a novel target for overcoming resistance in HER2-positive breast cancer (23).

In addition, recent studies have shown that the GPX4 inhibitor RSL3 can enhance chemosensitivity in luminal breast cancer by synergizing with other chemotherapeutic agents through ferroptosis (24). Buschhaus et al. further highlighted the role of iron metabolism in overcoming BCSC chemoresistance in both preclinical and clinical studies, proposing that combining iron modulation with targeted therapies could lead to favorable outcomes in luminal breast cancer (25). In conclusion, restoring the sensitivity of tumor cells to ferroptosis presents a promising approach to treating drug-resistant breast cancer. Notably, drug-resistant breast cancer cells often exhibit dependence on GPX4, suggesting that its inhibition could potentially overcome chemoresistance. Since cysteine serves as a substrate for glutathione synthesis and inhibits ferroptosis, cysteine depletion could restore chemosensitivity in breast cancer cells. Moreover, reducing cystine and GSH levels can induce ferroptosis, thereby inhibiting tumor growth.

## 4 Iron metabolism and chemotherapy resistance in breast cancer

### 4.1 Targeting ferritin or transferrin for chemoresistance treatment

Ferroptosis is characterized by elevated intracellular iron concentrations, which can further amplify the process through the generation of Lip-ROS. Iron metabolism plays a central role in ferroptosis, with the redox-active form of iron being crucial for the initiation of this process. Increased intracellular iron levels are a major contributor to chemotherapy resistance in breast cancer (26). Excess iron catalyzes the generation of ROS via the Fenton reaction, leading to oxidative stress and subsequent DNA damage. Iron overload is a key factor in promoting ferroptosis, and both iron autophagy and ferritin deposition are critical for this process (15). Zhu et al. highlighted that GATA3 can regulate iron metabolism and ferroptosis by inhibiting CYB5R2, thereby mediating resistance to doxorubicin in breast cancer (27). Moreover, iron chelators have been shown to inhibit ferroptosis, whereas transferrin and its receptor can activate ferroptosis (4).

Ferritin, the primary intracellular iron storage protein, plays a significant role in ferroptosis regulation. Upregulation of ferritin or its receptor has been observed in drug-resistant tumors, particularly in multi-drug-resistant breast cancers (26). Habashy et al. reported that the expression of transferrin receptor (CD71) was significantly increased at both mRNA and protein levels in endocrine-resistant breast cancer cells (28). Moreover, Bajbouj et al. suggested that the accumulation of intracellular iron contributes to chemotherapy resistance, and that iron efflux induced by transferrin could enhance doxorubicin sensitivity in estrogen receptor-positive (ER+) breast cancers (29). Further studies have shown that nuclear ferritin in breast cancer cells can protect DNA from damage caused by DNA-alkylating agents. Down-regulation of ferritin results in oxidative damage, thereby increasing drug sensitivity in tumor cells. These findings suggest that targeting ferritin or transferrin could represent a promising strategy to overcome drug resistance in breast cancer (30, 31). Moreover, gold nanoparticles loaded with polyherbal formulation and doxorubicin have been used to treat drug-resistant breast cancer cells. This approach enhanced ferroptosis through the degradation of ferritin, leading to significant antitumor effects (32).

### 4.2 Drugs and nanoparticles affecting iron metabolism in breast cancer

Iron metabolism in breast cancer is influenced by various chemotherapeutic agents. Yu et al. demonstrated that sulfasalazine could modulate iron metabolism through the transferrin receptor, thereby affecting ferroptosis in estrogen receptor-positive breast cancer cells. This finding presents a potential therapeutic strategy for breast cancer (33). Similarly, Ma et al. reported that in breast cancer cell lines treated with a combination of lapatinib and the lysosomal disruptor siramesine, intracellular iron levels could be significantly increased by upregulating transferrin or downregulating the transferrin receptor (34, 35). This process induces ferroptosis in tumor cells via Lip-ROS, which can be reversed by ferrostatin-1 and deferoxamine (DFO) (34).

Furthermore, recent studies have explored various nanotherapeutic systems or exosomes loaded with iron metabolism regulators and chemotherapeutic agents (e.g., doxorubicin and trastuzumab). These systems not only induce ferroptosis in breast cancer cells by modulating iron metabolism, but also enhance chemotherapeutic efficacy by increasing chemosensitivity, yielding promising results in cellular studies (36–39). For instance, Xiong et al. developed a drug-organic-inorganic self-assembled nano-system (DFTA) combining FeCl_3_ and doxorubicin for the treatment of estrogen receptor-positive breast cancer cells. This system significantly enhanced doxorubicin efficacy by promoting intracellular iron accumulation, depleting GSH, and inducing ferroptosis (40). Li et al. designed a novel nanoparticle comprising ferritin, erastin, and rapamycin, which induces ferroptosis by modulating GPX4 activity and demonstrates anti-breast cancer effects in a mouse model (41). Additionally, Yao et al. developed ferroptosis-promoting nanomedicine loaded with simvastatin to inhibit GPX4 in TNBC cells, highlighting a potential strategy to overcome chemotherapy resistance (42).

Exosomes, with their unique targeting capabilities and biocompatibility, offer distinct advantages in tumor therapy. Yu et al. engineered folate-labeled exosomes loaded with erastin for the treatment of TNBCs that overexpress folate receptors. These exosomes inhibited the Xc^-^ system, promoted Lip-ROS accumulation, and ultimately induced ferroptosis in MDA-MB-231 cells (43). Chen et al. applied a nano-system delivering chemotherapeutics (CPT), ferrocene (Fc), and the GPX4 inhibitor RSL3 to reduce chemoresistance in TNBC. The primary mechanism was ferroptosis induction via RSL3-induced GSH depletion, enhancing the efficacy of chemotherapeutic agents. These findings suggest that such drug formulations hold great promise for clinical application in TNBC, as summarized in **Table 1** (44).

**Table 1:** Potential antitumor agents inducing ferroptosis in breast cancer and their mechanisms of action.

Drug	Cancer type	Antitumor mechanism	Reference
AGuIX nanoparticles	TNBC	Regulate the anti-ferroptosis system by inhibiting the NRF2-GSH-GPX4 signaling pathway	(45)
Fe_3_O_4_@PCBMA-SIM nanosystem	TNBC	Inhibit the expression of HMGCR to downregulate the mevalonate (MVA) pathway and GPX4, thereby inducing cancer cell ferroptosis	(42)
ATO/SRF@BSA nanomedicine	TNBC	Induce augmented ferroptosis by depleting GSH and inhibiting DHODH activity	(46)
Cu_2-x_Se nanoreactor	TNBC	Generate O_2_ and consume intracellular GSH via the interconversion of Cu elements Cu^+^ and Cu^2+^, and impair the GPX4/GSH pathway and HIF-1α protein expression	(47)
Disulfiram/Copper	TNBC	Increase lipid peroxidation and cause a sharp increase in HMOX1 activity, thereby inducing TNBC cell death through ferroptosis	(48)
Combination therapy involving Danggui Buxue Tang and doxorubicin	TNBC	Modulate the Nrf2/HO-1/GPX4 axis	(49)
Sculponeatin A	Breast cancer	Promote the ETS1-SYVN1 interaction to induce ferroptosis in BC mediated by ETS1 degradation	(50)
Escin	Breast cancer	Inhibit tumor growth *in vivo* and *in vitro* via regulating the ferroptosis mediated by G6PD/GPX4 axis	(51)
Eupaformosanin	TNBC	Regulate mutant p53 ubiquitination	(52)
Yttrium Oxide nanoparticles	TNBC	Upregulate the pro-apoptotic genes CASP3 and CASP8 as well as ferroptosis-related gene HO-1	(53)
*Lycium barbarum* polysaccharide	Breast cancer	Prevent breast cancer cell proliferation and promote ferroptosis via the xCT/GPX4 pathway	(54)
Ketamine	Breast cancer	Suppress proliferation and induce ferroptosis by targeting KAT5/GPX4 axis	(55)
Shuganning injection	TNBC	Promote intracellular labile iron pool accumulation in an HO-1 dependent manner	(12)
Formononetin	TNBC	Induce ferroptosis by modulating the mTORC1-SREBP1 signaling axis	(56)
Trastuzumab	HER2-positive breast cancer	Circ-BGN could directly bind to OTUB1 and SLC7A11, enhancing OTUB1-mediated SLC7A11 deubiquitination and thereby inhibiting ferroptosis	(57)
Natural compound So-2	TNBC	Induce TNBC ferroptosis via downregulating the expression of E2F7	(58)
Curcumin	Breast cancer	Promote SLC1A5-mediated ferroptosis	(59)

## 5 Targeting System Xc^-^ to overcome chemoresistance in breast cancer

System Xc^-^, composed of solute carrier family members SLC7A11 and SLC3A2, is a crucial regulator of ferroptosis. Its expression is significantly elevated in breast cancer and various other drug-resistant tumors (60). System Xc^-^ can be activated by several stress conditions, including amino acid deprivation, electrophiles, oxidative stress, glucose starvation, and factors dependent on NRF2 and ATF4 (26). Additionally, the tumor suppressor P53 and BRCA1-associated protein 1 (BAP1) can modulate System Xc^-^ expression by inhibiting SLC7A11, thereby influencing ferroptosis (61). Sorafenib is a clinically approved anticancer drug and a highly potent inducer of ferroptosis, with System Xc^-^ contributing to sorafenib’s efficacy in drug-resistant cancers (62). The xCT subunit, a functional component of System Xc^-^, facilitates the exchange of intracellular glutamate for extracellular cysteine, a process essential for maintaining redox balance and supporting the survival of breast cancer cells. Hasegawa et al. showed that the transmembrane oncogene MUC1 C-terminal subunit (MUC1-C) targets xCT to preserve glutathione levels in TNBC cells. Silencing MUC1-C results in downregulation of xCT, triggering ROS-mediated ferroptosis and enhancing sensitivity to doxorubicin chemotherapy (63). Zhou et al. proposed that polyphyllin III induces ferroptosis via ACSL4 in TNBC cells, with chemotherapy resistance potentially being regulated by KLF4-mediated xCT expression (64). System Xc^-^ also mediates the effects of erastin in breast cancer, where it promotes ferroptosis and enhances the antitumor activity of cisplatin when combined with erastin under drug-resistant conditions. Furthermore, Li et al. demonstrated that in PARP inhibitor-resistant TNBC, resulting from poly (ADP-ribose) polymerase 1 (PARP1) gene mutations, chemotherapy resistance can be reversed by inducing ferroptosis through the P53/SLC7A11 signaling pathway, via proteolysis-targeted chimaera (PROTAC) (65). In conclusion, System Xc^-^ represents a promising target for overcoming drug resistance in breast cancer and may offer new therapeutic strategies for treating drug-resistant tumors.

## 6 Ferroptosis-associated oxidative stress pathway

The disruption of redox homeostasis is a critical pathway contributing to chemoresistance in cancer cells. It is well established that elevated GSH levels and reduced ROS generation in drug-resistant cells are key factors in acquired chemoresistance. Ferroptosis, a form of cell death driven by oxidative stress, is intricately linked to the regulation of redox balance. Not only does promoting oxidative stress facilitate ferroptosis, but it also helps overcome chemotherapy resistance. Recent studies have elucidated the impact of ferroptosis-related oxidative stress pathways on the sensitivity of several chemotherapeutic agents in breast cancer. One such pathway is the FSP1/CoQ10 axis, which has emerged as a critical regulator of ferroptosis, particularly in TNBC. FSP1 is a potent inhibitor of ferroptosis, functioning by reducing CoQ10 to ubiquinol, an antioxidant that protects against lipid peroxidation (66). This pathway operates independently of the GPX4/glutathione axis, providing an alternative mechanism for cancer cells to evade ferroptotic cell death. In TNBC, characterized by the absence of estrogen receptor, progesterone receptor, and HER2 expression, the FSP1/CoQ10 axis is pivotal in maintaining cellular redox homeostasis and promoting tumor cell survival. Targeting this axis has emerged as a promising therapeutic strategy to sensitize TNBC cells to ferroptosis-inducing agents (67). For instance, studies have shown that inhibiting FSP1 can synergize with GPX4 inhibitors to induce ferroptosis in TNBC cell lines, highlighting the potential for combination therapies (68). Additionally, FSP1 expression levels correlate with resistance to conventional chemotherapies, suggesting it could serve as both a therapeutic target and a biomarker for treatment response (69).

Valashedi et al. found that lipocalin 2 (Lcn2), a proteolipid transport protein with antioxidant properties, is upregulated in response to stress in multiple tumor types. In MDA-MB-231 breast cancer cells, Lcn2 inhibition was shown to promote erastin-mediated ferroptosis, leading to reduced tumor cell proliferation, metastasis, and enhanced chemosensitivity to cisplatin (70). These findings suggest that targeting Lcn2 to induce oxidative stress and enhance ferroptosis could offer a promising strategy for improving breast cancer treatment outcomes. Sun et al. reported that propofol could sensitize TNBC cells to doxorubicin and paclitaxel by promoting ferroptosis through the modulation of oxidative stress and the p53-SLC7A11-GPX4 pathway (71). Similarly, Song et al. demonstrated in both *in vitro* and *in vivo* studies that chemotherapy resistance to gefitinib in TNBC is associated with GPX4-mediated oxidative stress. Inhibition of GPX4 promoted ferroptosis and improved gefitinib sensitivity (72). Moreover, the underlying mechanism of doxorubicin resistance was found to be linked to oxidative stress, where elevated oxidative levels contributed to reduced tumor resistance. As one of the members of the ATP-binding cassette (ABC) superfamily, ABCC9 is elevated in TNBC samples and may serve as a potential biomarker in patients with TNBC disease (73). Collectively, oxidative stress targeting ferroptosis may also be an effective treatment for chemotherapeutic resistance in breast cancer.

## 7 RNA regulation in ferroptosis

In recent years, a growing body of research has shed light on the complex regulation of ferroptosis in breast cancer. Studies have identified a wide array of genes, proteins, and signaling pathways involved in this process. In addition to these, non-coding RNAs, including microRNAs (miRNAs) and long non-coding RNAs (lncRNAs), have also been implicated in the regulation of ferroptosis, further expanding our understanding of its molecular underpinnings (74). Clinical studies have highlighted the potential of ferroptosis-related genes as biomarkers for predicting treatment response. For example, genes such as acyl-CoA synthetase long-chain family member 4 (ACSL4) and GPX4 have been shown to serve as independent predictors of chemosensitivity in neoadjuvant chemotherapy for breast cancer, and their expression levels are strongly associated with patient prognosis (75). In a comprehensive study, Wu et al. identified 259 genes related to ferroptosis in breast cancer, 15 of which were found to be independent risk factors for prognosis. Additionally, they uncovered 1,185 ferroptosis-related lncRNAs and 219 ferroptosis-related miRNAs, broadening the scope of potential therapeutic targets in ferroptosis regulation (74). Further studies have focused on specific molecular players. Mao et al. discovered that the lncRNA P53RRA contributes to the ferroptosis process by regulating the expression of P53, which in turn plays a key role in mediating tumor drug resistance (76). Similarly, Wang et al. identified a novel circular RNA (circ-BGN) that is closely linked to trastuzumab resistance in HER2-positive breast cancer. Circ-BGN is overexpressed in resistant tumor cells, and its expression correlates with poorer patient survival. Importantly, knocking out circ-BGN restores chemosensitivity to tumor cells, a process that is mediated by erastin-induced ferroptosis (57). Taken together, these findings suggest that modulating ferroptosis could be a promising therapeutic strategy for overcoming trastuzumab resistance in HER2-positive breast cancer patients (57). By targeting key genes, non-coding RNAs, and regulatory pathways, it may be possible to improve treatment outcomes for patients who are resistant to current therapies.

## 8 Conclusion and discussion

Ferroptosis, a regulated form of cell death, plays a critical role not only in chemoresistance in breast cancer but also in the response to endocrine therapy, targeted therapies, immunotherapy, and radiotherapy (77). Despite its emerging significance, the underlying regulatory mechanisms of ferroptosis are not yet fully understood. Current research has largely concentrated on the regulation of iron metabolism and the System Xc^-^ pathway. However, further investigations are needed to comprehensively elucidate the molecular mechanisms of ferroptosis. Such studies could facilitate the development of targeted therapies aimed at ferroptosis, potentially reversing multidrug resistance in tumors.

Although several compounds have been identified as ferroptosis inducers, and novel drug delivery systems (such as exosomes and nanotechnology) are being explored to enhance drug delivery into cells, the clinical application of these strategies faces significant challenges. First, there remains a substantial gap in our understanding of the full regulatory network governing ferroptosis. Second, determining the optimal dosage of ferroptosis inducers for patients is crucial and requires further exploration. Third, potential crosstalk with other activation pathways, such as autophagy, may complicate the therapeutic targeting of ferroptosis and could lead to undesirable side effects. Finally, since ferroptosis occurs not only in tumor cells but also in normal tissues, its use in breast cancer therapy may result in unintended toxicity and serious complications.

Accumulating evidence suggests that the degree of ferroptosis can modulate sensitivity to various chemotherapy agents. For example, Zhu et al. demonstrated that in patients with breast cancer brain metastasis (BCBM), higher ferroptosis scores were associated with increased sensitivity to a wide range of chemotherapy drugs, including cisplatin, dasatinib, etoposide, gefitinib, lapatinib, pazopanib, sunitinib, docetaxel, and vinorelbine (78). Conversely, elevated ferroptosis levels were found to reduce sensitivity to drugs such as sorafenib, vorinostat, and doxorubicin. Similarly, Xu et al. highlighted that the expression of ferroptosis-related genes could influence the effectiveness of neoadjuvant chemotherapy and suggested that ferroptosis levels might serve as an independent prognostic factor. Specifically, high ferroptosis levels were linked to enhanced sensitivity to paclitaxel, gefitinib, doxorubicin, bleomycin, and bortezomib (79).

In conclusion, future research should focus on uncovering additional ferroptosis-related regulators and identifying biomarkers that can be used for patient stratification in breast cancer. To optimize clinical outcomes, it will also be crucial to develop rational treatment regimens, incorporate antioxidant therapies when appropriate, and monitor multiorgan functions to mitigate potential complications.
